# Knowledge, attitudes and practices regarding cervical cancer prevention at Thulamela Municipality of Vhembe District in Limpopo Province

**DOI:** 10.4102/phcfm.v8i2.1002

**Published:** 2016-06-17

**Authors:** Dorah U. Ramathuba, Doris Ngambi, Lunic B. Khoza, Nditsheni J. Ramakuela

**Affiliations:** 1 Department of Advanced Nursing Science, University of Venda, South Africa

## Abstract

**Background:**

Cervical cancer is a widespread and often fatal disease that affected 1 million women globally in 2005. Not only is it the second most common cancer in women but it was also the second leading cause of cancer deaths, accounting for over 250 000 in 2006.

**Purpose:**

The purpose of the study was to assess the knowledge, attitudes and practices regarding cervical cancer prevention among rural women in Vhembe District in Limpopo Province.

**Methodology:**

A quantitative descriptive approach was adopted. The quantitative design enabled the discovery of more information by means of direct questioning of a sample of women aged 30 years and older. A convenience sampling was used to select the respondents. Data were analysed using the Statistical Package for Social Sciences. Measures to ensure ethical issues were adhered to.

**Results:**

The findings revealed that women lacked knowledge about cervical cancer and preventive methods, and displayed positive attitudes to the use of services if made available as health workers did not adequately inform them about the availability of the services.

**Conclusion:**

The awareness of cervical cancer among women in Vhembe District is limited. There is a need to educate and promote awareness of cervical cancer among women to reduce the burden of morbidity and mortality.

## Introduction

Cervical cancer is a public health problem both in developed and developing countries although it is preventable and curable if identified at an early stage.^1^ Cervical cancer is the second most common cancer among women worldwide, but the commonest in developing countries. It accounts for approximately 12.0% of all cancers in women worldwide.^2^ The fact that cervical cancer screening is under-utilised and the mortality and morbidity rates of women due to cervical cancer remain high, is because women lack knowledge about human papilloma virus, vaccine and cervical cancer prevention as confirmed by.^3^

Throughout the world, prevention, control and treatment of cervical cancer have been a public health priority.^4^ The disparity in prevalences between the developing and developed world can be attributed to lack of awareness of cervical cancer and lack of effective cytological screening programs in developing countries.^5^ Other reasons include the constant focus on competing health priorities such as HIV and/or AIDS, tuberculosis and malaria.^2^ The world pattern of cervical cancer indicates that this is predominantly a problem of low-resource setting countries. The main reason is limited access to screening and treatment facilities. Cytological screening has been one of the most successful public health measures available for cancer prevention.^5^

Cervical cancer continues to be the leading cause of death for women in America. Incidence rate of cervical cancer in America is (8.9 per 100 000). More than 10 000 new patients develop cervical cancer each year, and 3600 women in the USA die from the advanced stage of this disease annually.^6^ Approximately 16 out of every 100 000 women in United States of America will develop cervical cancer and approximately 9 out of 100 000 will die from it.^6^

Sub-Saharan Africa has by far the highest burden and mortality associated with cervical cancer in the World.^7^ In less developed countries, this type of cancer is the second most common in women and accounts for up to 300 000 deaths annually, and 80% of cases occur in low-income or middle-income countries. Southern Africa has one of the highest incidence rates of deaths in the world.^8^ A total of 5318 new cases of cervical cancer were detected in South Africa in 1997, while the risk of development of cervical cancer in South African women has been estimated to be 1 in 29.^7^ In developing countries, lack of a population based screening programme accounts for women presenting in the advanced and often untreatable stage of the disease.^9^

Cervical cancer is a major problem in South Africa and it is the second most common cancer and affects one out of every 41 women.^9^ Women from poorer communities are most affected with this condition.^3^ In South Africa, current estimates are that 493 000 women are diagnosed with cervical cancer per year and 274 000 die from the disease.^10^ A study conducted in KwaZulu-Natal revealed that cervical cancer was identified as health priority.^8^ In Limpopo estimates for cervical cancer deaths in 2000 were 5.6%.^11^

The South African Department of health launched the national guideline on cervical cancer screening programme to reduce the incidence and the burden of cervical cancer. The aim of the programme is to screen all women over 30 years three times in their lifetime at a 10 year interval^9^ and to reduce mortality and morbidity from cervical cancer and decrease the number of patients suffering from the disease.^12^ According to the National Health Plan for South Africa, the high maternal mortality rates are a great concern, especially among the disadvantaged. A key focus of the policy is improving the health status of women and ensuring that mechanisms for the mother, women youth and adolescent (MCWH & Y) are created so that no mother dies because of lack of access to health services.^13^

## Aim of the study

The aim of the study is to determine the knowledge, attitudes and practices regarding cervical cancer prevention among rural women in Thulamela Municipality at Vhembe District in Limpopo Province.

The objectives of the study were:

- To assess the knowledge of rural women regarding cervical cancer and preventative measures.
- To describe the attitudes and practices of cervical cancer prevention.
- To recommend ways to improve screening uptake.

### Contribution to the field

The study has added to the already existing body of knowledge about cervical cancer prevention strategies. Primary health care services need to improve on the strategies to disseminate information regarding cervical cancer screening, prevention, treatment and benefits by marketing the service and disseminating pamphlets in local languages. The reduction of morbidity and mortality associated with cervical cancer will reduce the expenditure on the treatment of invasive cancer. Therefore, intense knowledge dissemination and access to preventive measures will increase provincial targets and reduce the burden of cervical cancer and mortality.

### Methodology

A quantitative, cross-sectional descriptive design was used to assess and describe knowledge, attitudes and practices regarding cervical cancer prevention among rural women aged 30 years and older in Vhembe District in Limpopo Province. Target population is the aggregate of cases about which the researcher would like to make generalisations.^14^ The target population was all women who came for consultation in four selected clinics. Elements are selected by non-random methods.^15^ Four local areas under Thulamela A and B were selected purposefully based on the two hospitals’ gynaecological register reports of 30 mortalities in 2013 and 2014 and the referring clinics were purposefully sampled. Participants were sampled through convenience sampling method, the sample size was 1546. Data were collected at the respective clinics on a particular day that the researcher visited the clinic per the convenience of the participants who were awaiting consultation. Participants answered coded questions on the self-administered questionnaire that assessed their knowledge, attitudes and practices regarding cervical cancer screening. Data entry and analyses were undertaken using the computer software Statistical Package for Social Sciences version 22. In the analysis, appropriate frequencies were generated and descriptive results were presented. The results were grouped under knowledge, attitudes and practice areas. Differences between categorical variables were analysed using the chi-square test, and *p*-values of less than 0.05 were considered significant.

## Results

### Knowledge regarding cervical cancer prevention

This subsection deals with data on the knowledge regarding cervical prevention. The following were used to gather data from the respondents (see Table 1).

**TABLE 1:** Participants’ characteristics.

Variable	*N*	%
**Age**		
30–35 years	336	21.7
36–40 years	490	31.7
41 and above	720	46.6
**Marital status**		
Married	886	57.3
Single	488	31.6
Widowed	112	7.2
Divorced	60	3.9
**Ethnicity**		
Venda	1066	69.1
Tsonga	296	25.6
Sotho	84	5.3
**Education level**		
Grade 7	588	36.1
Grade 11	600	38.8
Completed grade 12	248	16.0
Never attended school	140	9.1
**Employment status**		
Unemployed	1229	83.6
Employed	254	16.4
**Parity: Number of children**		
Null parity	0	2.8
-	1	8.5
-	2	25.5
-	3	21.9
-	4	23.4
-	5	14.5
	6	3.4

*Source*: Authors’ own work

### Knowledge of cervical cancer

**FIGURE 1:**
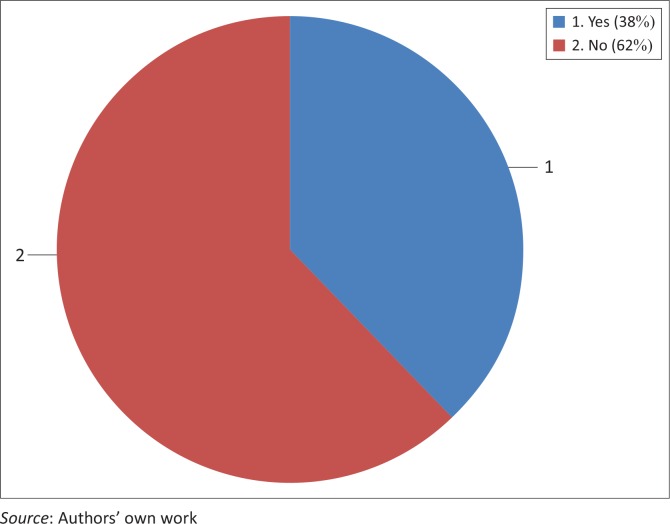
Distribution of respondents on knowledge about cervical cancer (*n* = 1546).

The results show that the majority of the respondents, 62.0% (958), indicated that they had never heard about cervical cancer while 38.0% (588) indicated that they had heard about cervical cancer (see Figure 1).

These results show that the majority 62.0% of women aged 30 and above were not aware of cervical cancer and only 38.0% were aware of cervical cancer (see Table 2).

**TABLE 2:** Willingness of participants to take a pap test (Chi-square tests for selected variables).

Pair of variables tested	Pearson Chi-square value	Df	Asymp. Sig. (2 sided) (*p*)	0 cells (0.0%) have expected count less than 5
Age range cervical cancer knowledge cross-tabulation.	10.641	2	0.005	The minimum expected count is 83.89
Highest level of education attained Knowledge of cervical cancer.	31.137	3	0.000	The minimum expected count is 34.95
Worriedness of getting cancer highest level of education attained.	23.229	9	0.006	The minimum expected count is 2.45

*Source*: Authors’ own work

The chi-square results show a significant relationship between level of knowledge of cervical cancer and the age range of respondents; *X*^2^ (2, *N* = 1546) = 10.641, *p* < 0.005. Knowledge of cervical cancer was associated with age range. The results show that 46.6% of respondents aged between 30 and 35 were more likely to have knowledge of cervical cancer compared with 31.7% of respondents aged between 36 and 40 years and 21.7% of women aged 41 and above. The correlation of age and respondents’ knowledge revealed that as the age range increased the knowledge about cervical cancer decreased, *r* = -0.671 at *p* < 0.01. This implies that age range was an influential factor with regard to the respondents’ knowledge about cervical cancer. Young women were likely to have more knowledge on cervical cancer than elderly women.

**FIGURE 2:**
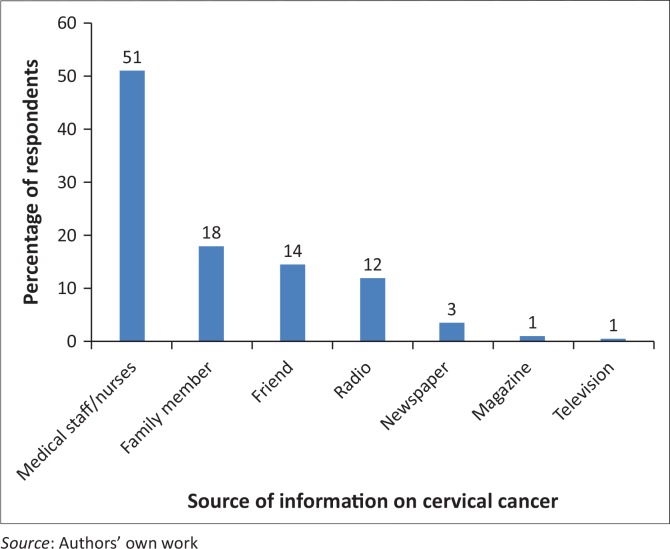
Distribution of respondents according to sources of information on cervical cancer.

Figure 2 shows the distribution of respondents with respect to the source of information.

Content of information about cervical cancer: A follow-up was made on the content of information about cervical screening that the respondents had received. The results show that the content information was relevant to cervical cancer; however, only 15.8% (244/1546) of the whole sample indicated that the content information was relevant to cervical cancer.

On cervical cancer risks factors: The results show that the minority of the sample had some knowledge about risk factor for cervical cancer (see Table 3).

**TABLE 3:** Cervical cancer risk factors.

Risk factors	Yes		No		Total	
		
	Frequency	%	Frequency	%	Total	%
Multiple sex partners	203	35	385	65	588	100
Having genital virus	189	32	399	68	588	100
Sexual intercourse before 18	200	34	388	66	588	100
Having contracted STIs	213	36	375	64	588	100
Smoking cigarettes	228	39	360	61	588	100
Use of oral contraceptives	158	27	430	73	588	100

*Source*: Authors’ own work

The results show that only 6.1% (97) of the respondents indicated that cervical cancer could be diagnosed using the Pap smear, 12.2% (188) indicated biopsy and 19.4% (302) indicated blood tests. These results indicate that in this sample, 18.2% (282) of the respondents knew the diagnosis of cervical cancer while the majority 81.8% (1264) of the respondents did not know the diagnosis of cervical cancer (see Table 4).

**TABLE 4:** Distribution of respondents on preventability of cervical cancer and taking Pap smear and Pap smear offered at local clinic.

Variable	Yes		No		Do not know		Total	
			
	Frequency	%	Frequency	%	Frequency	%	Total	%
Preventability of cervical cancer	250	16.2	101	6.5	237	15.3	1546	100
Taking Pap smear	50	3.2	1496	96.8	0	0	1546	100
Pap smear offered at local clinic	132	8.5	358	23.2	1056	68.3	1546	100

*Source*: Authors’ own work

The results show that only 50 respondents (3.2%) took a Pap smear and that 1496 (96.8%) did not. This points to the lack of awareness of cervical cancer and lack of knowledge on the importance of taking the screening.

Table 5 shows the findings on the possibility of respondents getting a test done when given a pamphlet about Pap smear.

**TABLE 5:** Distribution of respondents on the willingness to take a cancer screening test.

Response	Frequency (*n* = 1546)	%
Do not know	264	17.1
No	348	22.6
Yes	930	60.3

**Total**	**1542**	**100.0**

*Source*: Authors’ own work

The results show that 264 (17.1%) respondents were not decided about taking a cancer screening test and majority were not keen even when given a pamphlet about Pap smear. These results are an indication of the negative effects of lack of information on cervical cancer.

## Discussions

Cervical carcinoma is still the most common cancer among women in the African continent. Mortality remains high worldwide at 50% mainly because of late presentation, advanced stage of the disease and absence of a functioning screening process.^12^ Cervical cancer is a preventable disease, and a key aspect of its prevention is the detection of the premalignant lesion by cervical screening. The major challenge for the study is the high percentage (62.0%) of low levels of knowledge about cervical cancer, which is unacceptable as the majority of women had secondary education, meaning that they were literate:^16^ ‘found a positive relationship between education and cervical cancer preventive measures.’ Furthermore data revealed that 48.5% (285/1546) of the respondents knew the diagnosis of cervical cancer while the majority 51.5% (1231/1546) of the respondents did not know the diagnosis of cervical cancer. The study concurs with the findings of Hoque and Hoque^10^ that ‘female university students also lacked information about cervical cancer screening practices’. Perkins et al.^17^ reported that ‘usually women with high education may not necessarily seek screening, thus additional factors must be considered’. Further analysis of awareness about cervical cancer being preventable revealed that only a minority, 16.2% (250/1546), of the whole sample were aware that cervical cancer was preventable and the majority were not aware of the risks factors. Regarding sources of information on cervical cancer, majority indicated nurses followed by friends, and the media was less cited. Nurses are said to be at the forefront of primary education, and women who have accessibility to nurses are likely to have knowledge about cervical cancer.

The results show that knowledge about cervical cancer is fairly and negatively related to the age range of respondents. As the age range increased, the knowledge about cervical cancer decreased, *r* = -0.671 at *p* < 0.01. This implies that age range was an influential factor on the respondents’ knowledge about cervical cancer. Young women were likely to have more knowledge on cervical cancer than elderly women. There is a need to target these young groups so as to increase their level of knowledge and curb future morbidities and expel myths and beliefs that cervical cancer does not affect older women. There is a critical need for the primary health nurses, private sector and government to increase the level of knowledge and awareness of cervical cancer and screening methods among women and also provide screening facilities. Educational campaigns are important in improving knowledge about cervical cancer prevention and promote acceptance of Pap smear screening.^18^ Furthermore to make the most sustainable programme possible, Nwankwo et al.^18^ suggest that ‘local health providers and other community members should work together to develop education programmes that addresses the knowledge deficit in communities.’ The study findings further revealed that only 50 respondents (3.2%) took a Pap smear and 1496 (96.8%) did not. This is suggestive of the lack of awareness of cervical cancer and the lack of knowledge on the importance of screening. Reasons cited by participants reflected ignorance on part of the participants as 44 (36.1%) respondents indicated that there was no reason for not taking a Pap smear, 38 (31.1%) indicated fear as a reason of not taking a Pap smear, 27 (22.1%) indicated that they did not have information on Pap smear while 13 (10.7%) cited time factor. This is supported by a Mexican study that revealed that ‘the reason for women not obtaining pap smear included anxiety regarding physical privacy, lack of knowledge and difficult accessing health care and also reported fear that any gynaecological treatment would leave them sexually disabled’.^19^ Another study conducted among Chinese American immigrants reported that ‘women believed that women did not need Pap test if they had no symptoms, were not having intercourse with a man and were post-menopausal’.^20^ Regarding their attitudes to taking a Pap smear if provided with a pamphlet, the results indicated that probably lack of information on cervical cancer was a cause for not taking a screening as 264 (17.0%) were undecided and 348 (22.6%) were not keen. The results on Pap smear knowledge and worriedness shows a weak and negative association of age range. Knowledge on Pap smear tend to decrease with age range *r* = -0.263, *p* < 0.01. This means that most of the elderly people have never heard about Pap smear compared with younger women. The knowledge about Pap smear also associates positively with the highest standard of education attained, *r* = 0.184 and *p* < 0.01. Results also show that worriedness associates negatively with age range and the highest standard of education achieved, *r* = -0.184, *p* < 0.01., which implies that young women are worried of contracting cervical cancer than older women. Worriedness also associates with the highest standard of education attained, *r* = 0.131, *p* < 0.01; the higher the level of education the more likely the women are worried about contacting cervical cancer. It is therefore necessary to target the older age groups in cervical cancer campaigns and to increase cervical cancer uptake as the incidence of cervical cancer increases with age, sensitising women about the risk factors such as parity since most participants reported parity of two to five. Other risk factors such as the use of oral contraceptives 430 (73.0%), genital warts 399 (68.0%) and early sexual intercourse 388 (66.0%) were suggestive of the fact women lacked knowledge about these risk factors. Lack of awareness of related risk factors and screening measures delays early and prompt diagnosis; this is also confirmed by^21^ who reported that: ‘black women presented late with advanced stage of disease due to unfavorable lack of cancer awareness and knowledge among the black population’. The findings from this study have important implications for health practitioners. Nurses are the backbone of primary health care, especially in rural areas, and they should be at the forefront of making the services available to the communities by informing and encouraging women to make use of the screening services. If health workers are knowledgeable and are pro-prevention, they will encourage women to use the screening services.

## Limitations

The study findings are not transferable to other settings as the study was conducted only in one district and only limited primary healthcare facilities were utilised. The study employed only one method of data collection on knowledge, attitudes and practices of cervical cancer prevention using close coded instrument which may have yielded more information if triangulated.

## Recommendations

Health promotion campaigns need to ensure that all women, particularly older women, continue to be educated on cervical cancer as well as the importance of having regular Pap smear test.

Providing women with informational pamphlets written in the indigenous languages outlining the risks and benefits:

1. Health education about cancer should be encouraged at social and cultural gatherings.
2. Primary healthcare nurses at local clinics should promote the uptake of cervical cancer screening.
3. Multi-sectoral approach in education with participation by private sector/NGOs, government, community groups and faith-based organisations.

## Conclusion

The knowledge on cervical cancer and preventive screening among rural women is lacking in Vhembe District (see Appendix 1). Knowledge increases the worriedness of having cancer and increases the uptake of having a Pap smear than those having no information. There is a need to educate and promote awareness among rural women regarding cervical cancer screening and prevention. Educating women at an early age through to the menopausal age and promoting awareness about risk factors and prevention measures will curb maternal mortality rates and improve the quality of lives of women. Targeting older women is of utmost significance as the results revealed that older women lacked information about cervical cancer and the preventive measures.

## Appendixes

### Appendix 1 Knowledge on Cervical cancer

#### Section B: Knowledge regarding cervical cancer prevention

1. Have you ever heard about cancer of the cervix? (Please circle)
  **Table:**
  Yes
  No
2. If yes, source of information (Please circle, you may circle more than one)
  - Friend
  - Family member
  - Medical or nursing personnel
  - Other………. (Please circle) (a) Magazine (b) TV (c) Radio (d) Newspaper
3. What was the content of information about cervical screening you received?
  ……………………………………
4. Risk factors for cervical cancer can be:
  **Table:**
  Multiple sex partners	Yes	No
  Having genital warts	Yes	No
  Sexual intercourse before 18	Yes	No
  Having contracted STIs	Yes	No
  Smoking cigarettes	Yes	No
  Use of oral contraceptive	Yes	No
5. Cervical cancer can be diagnosed by:
  - Pap smear
  - Biopsy
  - Taking blood
  - None of the above
6. Is cancer of the cervix preventable? (Please circle) Yes/No/Do not know
7. If Yes, which of the following are preventive measures for this type of cancer? (Please circle, more than one if you like). If No, do not answer
  - Using condoms
  - Good hygiene
  - Pap smear
  - Human papilloma virus (HPV) vaccine
  - Not smoking
  - Exercise
  - Delaying age of first sexual contact
8. Have you ever heard about screening tests for cervical cancer?
  **Table:**
  Yes
  No
9. What is it called?……………………………………………………………….
10. Have you ever had a Pap smear?
  **Table:**
  Yes
  No
11. Did your clinic offer it to you?
12. What was the reason for not having the test done?
13. If you were given a pamphlet about the Pap smear, would you do the test?
  **Table:**
  Yes
  No
  Do not know

#### Section C: Knowledge about human papilloma virus (HPV)

14. Have you ever heard of HPV? (Please circle) Yes/No
  (If your answer is Yes, proceed. If No, stop)
15. Who/What was the source of the information regarding HPV? (Please circle, circle more than one if you wish)
  - Parent
  - Neighbour/friend
  - Family member
  - Medical or nursing staff
  - Other (Please circle) (i) Magazine (ii) TV (iii) Radio (iv) Newspaper
16. The virus associated with cervical cancer is transmitted by:
  - Sexual intercourse
  - Kissing
  - Not using a condom during sexual intercourse
  - None of the above
17. HPV can cause cervical cancer (Please circle) True/False/Do not know
18. Who can become infected with HPV? (Please circle)
  - Men only
  - Women only
  - Both men and women
  - Do not know
19. Most people with genital HPV do not have symptoms of the infection (Please cross)
20. How worried are you of getting HPV? (Please cross)
  **Table:**
  (a) Not worried
  (b) A little worried
  (c) Very worried
21. If you were given a pamphlet about Pap smear and vaccine for HPV, would you do the test?
  **Table:**
  Yes
  No
  Not sure
